# Vitamin D in Pain Management

**DOI:** 10.3390/ijms18102170

**Published:** 2017-10-18

**Authors:** Maria Helde-Frankling, Linda Björkhem-Bergman

**Affiliations:** 1 ASIH Stockholm Södra, Långbro Park, Palliative Home Care and Hospice Ward, Bergtallsvägen 12, SE-125 59 Älvsjö, Sweden; maria.helde.frankling@ki.se; 2 Department of Laboratory Medicine, Division of Clinical Microbiology, Karolinska Institutet and Karolinska University Hospital, Huddinge, SE-141 86 Stockholm, Sweden

**Keywords:** vitamin D, pain, opioid, infections, quality of life, cancer, statins, myopathy

## Abstract

Vitamin D is a hormone synthesized in the skin in the presence of sunlight. Like other hormones, vitamin D plays a role in a wide range of processes in the body. Here we review the possible role of vitamin D in nociceptive and inflammatory pain. In observational studies, low vitamin D levels have been associated with increased pain and higher opioid doses. Recent interventional studies have shown promising effects of vitamin D supplementation on cancer pain and muscular pain—but only in patients with insufficient levels of vitamin D when starting intervention. Possible mechanisms for vitamin D in pain management are the anti-inflammatory effects mediated by reduced cytokine and prostaglandin release and effects on T-cell responses. The recent finding of vitamin D-mediated inhibition of Prostaglandin E2 (PGE2) is especially interesting and exhibits a credible mechanistic explanation. Having reviewed current literature, we suggest that patients with deficient levels defined as 25-hydroxyvitamin D (25-OHD) levels <30 nmol/L are most likely to benefit from supplementation, while individuals with 25-OHD >50 nmol/L probably have little benefit from supplementation. Our conclusion is that vitamin D may constitute a safe, simple and potentially beneficial way to reduce pain among patients with vitamin D deficiency, but that more randomized and placebo-controlled studies are needed before any firm conclusions can be drawn.

## 1. Introduction

Vitamin D is a hormone mainly synthesized in the skin in the presence of sunlight. Oral intake from natural and fortified foodstuffs, as well as from supplementation, adds to vitamin D levels in the individual. Like other hormones, vitamin D plays a role in a wide range of processes in the body. Sufficient vitamin D levels are important not only for a healthy skeleton but also for a healthy immune system [1]. Vitamin D has anti-inflammatory effects in the body by reducing the release of pro-inflammatory cytokines and suppressing T-cell responses [1,2]. In vitro studies have shown that vitamin D inhibits the synthesis of Prostaglandin E2 (PGE2) [3]. Both observational and interventional studies suggest a role for vitamin D in pain intensity and in management of pain in varying clinical settings [4,5,6,7,8,9,10]. Despite these data, three meta-analyses of randomized and placebo-controlled trials (RCTs) could not establish a correlation between vitamin D supplementation and pain reduction [8,11,12]. In contrast, a recent systematic review of published RCTs (not included in the three meta-analyses above) concluded that vitamin D supplementation leads to a significantly greater mean decrease in pain score compared to placebo in patients with chronic pain [13]. The goal of this review is to examine the evidence to date in the field of vitamin D and pain, and to elucidate whether current knowledge supports vitamin D supplementation in patients with deficient levels of 25-OHD and pain.

The search strategy in PubMed included the MeSH terms “vitamin D” and “pain” and resulted in 347 titles that have been briefly reviewed and relevant articles have been extracted in full text.

## 2. Definitions of Pain

The definition of pain, according to the International Association for the Study of Pain, is “… an unpleasant sensory and emotional experience associated with actual or potential tissue damage, or described in terms of such damage.” [14]. Physical pain is usually divided into nociceptive, inflammatory and neuropathic pain. During recent years different types of psychological pain has been defined, and “existential pain” is sometimes used to describe this feeling of pain in cancer patients [15]. Patients often experience “mixed pain”, where nociceptive, inflammatory and neuropathic components present together with existential pain. Nociceptive pain can be treated with opioids and non-steroid anti-inflammatory drugs (NSAIDs), whereas steroids and NSAIDs are used to treat inflammatory pain. Neuropathic pain can be treated with subgroups of anti-epileptic drugs or tricyclic antidepressants. Studies have shown that vitamin D might influence nociceptive and inflammatory pain mechanisms [4,6,9,16]. There are very scarce data on the role of vitamin D in neuropathic pain. One observational study has shown possible associations between vitamin D deficiency and carpal tunnel syndrome [17], and an interventional study showed positive effect on diabetic neuropathy after a very high single-dose of vitamin D [18]. However, in this review we will focus on the role of vitamin D in nociceptive and inflammatory pain.

## 3. Vitamin D—Synthesis, Physiological Effects and Pathophysiological Mechanisms

Vitamin D is synthesized in the skin using energy from ultraviolet-B light. Vitamin D is hydroxylated in two steps into the active form 1,25-dihydroxyvitamin D, which binds to the vitamin D receptor (VDR). The activated VDR complex regulates a large number of genes [19]. The pro-form 25-hydroxyvitamin D (25-OHD) has a half-life of about 3 weeks and is more stable than the active 1,25 hydroxy vitamin D, with a half-life of four hours [20]. Therefore, 25-OHD is used for evaluation of vitamin D status [21]. Serum levels of 25-OHD below 50 nmol/L are considered insufficient according to the Institute of Medicine in the US [22].

Vitamin D is needed for a healthy skeleton and plays an important role in calcium homeostasis. During recent years, it has become evident that vitamin D, just like other hormones, affects a variety of functions and processes in the body.

In our research we mainly focus on the role of vitamin D in the immune system. Vitamin D influences both the adaptive and the innate parts of immunity. For example, vitamin D is a potent inducer of antimicrobial peptides (AMPs) on mucosal surfaces and in immune cells [1,2]. AMPs constitute the “first line of defense” for invading bacteria and viruses on mucosal surfaces, including the respiratory tract [1,2]. In a meta-analysis comprising 25 randomized, placebo-controlled trials, including 11,321 participants, vitamin D supplementation was shown to reduce the number of respiratory tract infections [23]. This meta-analysis also showed that the protective effect of vitamin D was evident only in studies using once-daily dosage, whereas bolus doses did not work. Moreover, the positive effect was more pronounced in patients with serum levels of 25-OHD below 25 nmol/L. Importantly, vitamin D supplementation was generally well tolerated and not associated with any adverse events in any of the included studies.

In addition to the induction of AMPs, vitamin D also affects T-cell responses and suppresses inflammation [24]. Under conditions of vitamin D insufficiency, the immune system will favor a more inflammatory immune response involving Th1 and Th17 cells rather than Th2 and Tregulatiry cells (Treg) [24]. On the contrary, adequate levels of vitamin D lead to less inflammation and lower levels of inflammatory cytokines and prostaglandins [6]. Indeed, vitamin D deficiency has been reported as a risk factor for different forms of inflammatory diseases, such as rheumatic diseases [16,25] and multiple sclerosis [26,27,28]. Effects of vitamin D on both B- and T-cell responses have been suggested to be involved in controlling inflammatory diseases [16,27]. Colotta et al. recently published a valuable overview of the effects of vitamin D on immune and inflammatory cells and of epidemiological studies in the field [29]. The effects of vitamin D in the immune system that may influence pain management are listed in Table 1.

The physiological mechanism linking vitamin D to pain is not yet fully elucidated. Evidence from both clinical and animal studies suggest that insufficient levels of vitamin D affect both peripheral [30,31] and parasympathetic nerve function [32]. Presence of vitamin D receptors (VDR) and vitamin D activating enzymes in the central nervous system (CNS) as well as effects of vitamin D on neurotransmitters have been suggested to explain the link between pain and vitamin D in patients with fibromyalgia [33].

However, the most probable mechanism of Vitamin D in pain management involves its anti-inflammatory effects. As mentioned above, vitamin D shifts the T-cell responses resulting in higher levels of Th2 and Treg cells instead of the pro-inflammatory Th1 and Th17-cells [24]. In addition, in vitro studies have shown that vitamin D inhibits the synthesis of PGE2 in fibroblasts [3]. In a small study on 36 healthy women, vitamin D supplementation led to decreased levels of prostaglandins in a dose-dependent fashion [34]. Interestingly, in a clinical study where vitamin D supplementation reduced musculoskeletal pain, the effect was associated with decreased levels of inflammatory cytokines including prostaglandin E2 (PGE2) [6]. PGE2 is an important factor in inflammatory pain [35]. Thus, the suppression of inflammation in general and of PGE2 especially exhibits credible mechanistic explanations for the effect of vitamin D in pain.

Nevertheless, it can also be argued that vitamin D does not have any direct or causal role in pain. In fact, a recent article suggested that UVB light has strong analgesic effects by induction of endogenous opioid-like substances (endorphins) in the skin [36]. In this model, vitamin D levels would only serve as a maker for UVB exposition. Thus, it is possible that findings in observational studies of a clear association between vitamin D levels and pain can be fully or partly explained by exposure to the sun and endogenous synthesis of endorphins. However, if UVB light constituted the only association between vitamin D deficiency and pain, it would be hard to explain the positive effect on pain management reported during vitamin D supplementation [9,37,38,39,40].

## 4. Vitamin D and Chronic Pain

Chronic pain is generally described as pain experienced on a majority of days for at least three months. Several observational studies have shown that patients with different forms of chronic pain have low 25-OHD levels in the circulation [41,42,43,44]. This has led to that several randomized controlled trials in different populations of chronic pain patients have been conducted. In 2015, a Cochrane review was published with the aim to assess the efficacy and safety of vitamin D supplementation in patients with chronic pain. In the final analysis 10 studies, comprising a total of 811 patients, were included. In these studies vitamin D was compared to placebo or active comparators [12]. However, the studies were very heterogeneous regarding doses used, included patients and co-interventions. The authors of the Cochrane review concluded that there was no consistent pattern that vitamin D supplementation was associated with greater efficacy than placebo. However, it should be noted that vitamin D supplementation was considered safe and well tolerated, since the numbers of adverse events were the same between placebo groups and vitamin D-treated groups [12]. A recent review discussing the role of vitamin D in the treatment of chronic pain arrives at the same conclusion as the Cochrane review, arguing that in the absence of a mechanistically explanation for the causal relationship between vitamin D and chronic pain, clinical data from interventional studies do not support vitamin D supplementation as an independent treatment in this patient group [45]. The most promising results from clinical studies in patients with chronic pain were reported in patients suffering from fibromyalgia [9] or nonspecific musculoskeletal pain with insufficient 25-OHD levels at baseline [39], discussed further below. In contrast, in the study where the included patients had high/sufficient 25-OHD levels from the beginning (100 nmol/L), no effect of vitamin D supplementation was evident [46].

## 5. Vitamin D in Musculoskeletal Pain

Observational studies have indicated that sufficient vitamin D levels are important for normal muscular function and strength [47]. In addition, several studies have shown an association between vitamin D levels and neuromuscular coordination [47]. However, the presence of the vitamin D receptor (VDR) in adult skeletal muscle cells has been debated. Studies from VDR knock-out mice have shown that the muscle fibers were small and variable in size, although overall myocyte differentiation occurred normally [48]. Recently it was shown that the vitamin D-activating system can be detected in human muscle precursor cells, but is low or non-detectable in adult skeletal muscle [49]. Neither did a review of bioinformatics approaches in analyzing VDR signaling reveal a significant role for VDR in muscle tissue [50].

Fibromyalgia is a disease associated with chronic muscular pain [9]. In a small, randomized, placebo-controlled study (*n =* 30) vitamin D supplementation of 50,000 IU/week for 20 weeks showed statistical significant improvement in pain and quality of life [9]. The included patients had mean 25-OHD levels of 50 nmol/L at baseline. In a more recent, small, non-controlled interventional study (*n =* 58) on patients with chronic nonspecific and widespread musculoskeletal pain and mean 25-OHD levels of 20 nmol/ L at baseline, vitamin D supplementation with the same dose, 50,000 IU/week for three months, also showed a statistical significant improvement in pain and quality of life [51]. In contrast, in a study on patients with diffuse musculoskeletal pain with sufficient 25-OHD levels (mean 72 nmol/L) at baseline, no effect could be observed [52].

In a randomized, placebo-controlled study on 80 patients with musculoskeletal pain, 4000 IE vitamin D/day for 3 months resulted in improvement of symptoms recorded as decline in visual analogues scale (VAS) score [6]. The included patients had mean 25-OHD levels of 55 nmol/L at baseline. Interestingly, this study also showed that vitamin D supplementation resulted in decreased levels of inflammatory and pain-related cytokines in plasma, such as Prostaglandin E2 (PGE_2_), TNF α and Leukotriene B_4_ (LTB_4_) [6].

Low vitamin D levels have been linked to an increased risk for statin induced myalgia in several observational studies [7,53,54]. Interventional studies have shown that supplementation with vitamin D with 50,000 IU/week, may have protective effects against myalgia in statin treated patients [55,56]. However, these studies were not randomized or placebo controlled, compromising the validity of the data. Notably, several observational studies have failed to establish a relationship between low vitamin D levels and myalgia [57,58,59]. Thus, the picture is far from complete and further research is highly warranted.

## 6. Vitamin D in Cancer Pain

Several observational studies show that cancer patients generally have lower vitamin D levels than healthy controls [60,61,62,63]. According to two small pilot-studies, vitamin D supplementation might reduce pain in prostate cancer patients with bone metastases [37,40]. The association between vitamin D deficiency and musculoskeletal symptoms induced by aromatase inhibitor treatment in breast cancer patients has also been studied in different settings [64,65,66]. However, in a recent randomized, placebo-controlled trial on 160 breast cancer patients treated with aromatase inhibitors (the VITAL study) vitamin D supplementation was not associated with any clear beneficial effects on musculoskeletal symptoms [67].

In an observational study in palliative cancer patients in Stockholm, we noted an association between low 25-OHD levels and high opioid doses [4]. In a follow-up study, vitamin D supplementation was given in a dose of 4000 IE/day to palliative cancer patients with 25-OHD levels <75 nmol/L [38]. In this study we could show that the vitamin D-supplemented patients had significantly decreased fentanyl doses compared to untreated controls already after one month. The mean difference in fentanyl dose was 46 µg/h; (95% confidence interval 24–78 µg/h) between the groups after one month (*n =* 39) and increased even further at three months to 91 µg/h (95% CI 56–140 µg/h) (*n =* 26) [38]. In addition, there was a decrease in infections after three months of vitamin D supplementation as well as an increase in self-assessed quality of life. The results from this pilot study were used for the power-calculation of a future randomized, placebo-controlled, double-blind study called “Palliative-D” that will start in November 2017 and will include *n =* 254 palliative cancer patients [68]. The primary endpoint in that study will be “change in opioid dose” between placebo and vitamin D-treated patients. The study will proceed for two years and end in December 2019.

## 7. Which Dose Should Be Given to the Vitamin D-Deficient Patient?

When evaluating results from clinical vitamin D trials, it might be of value to consider the five rules for optimizing individual clinical studies of nutrient effects that Heaney et al. proposed in 2014, namely that both the basal and the change in nutrient status be measured so that a change in nutrient status produces the effect tested in the hypothesis, and that the intervention must be large enough to change nutrient status [69]. Also, changes in conutrient status should not influence the results of the study. Not all performed studies fulfill these rules—most often by not recording the individual patient’s baseline levels of 25-hydroxyvitamin D (25-OHD) [70]. If patients with sufficient vitamin D levels at baseline are included in an intervention study, they will not benefit from supplementation. This is often forgotten when vitamin D studies are evaluated and compared. Thus, the goal of vitamin D supplementation should be to achieve sufficient levels of 25-OHD in the circulation.

Serum levels below 50 nmol/L are considered to be insufficient according to the Institute of Medicine in the US [22]. However, based on the findings from recent clinical studies, it has been suggested that levels above 75 nmol/L might be the goal of supplementation [71,72,73]. Levels above 125 nmol/L probably add no more benefit, and levels above 250 nmol/L are considered as potentially harmful [74].

The recommended dietary intake (from both foodstuffs and supplementation) of vitamin D from all sources varies between 400–600 IU/day in different countries [74]. However, when supplementing individuals with vitamin D deficiency, a total oral intake in this range does not result in a quick change in vitamin D status. In accordance with Heaney’s principles mentioned above, the intervention needs to be large enough to change nutrient status. In individuals with serum levels of 25-OHD <30 nmol/L we therefore suggest supplementation with 4000 IE /day for at least three months, or until the levels are >50 nmol/L. This dose has been shown to be both effective and safe in clinical studies [23,38,75]. This dose has also been stated as safe in adults according to guidelines from expert groups [74]. The safety issue has been further addressed in a large meta-analyses including 11,321 and 821 participants respectively; vitamin D supplementation was never associated with more adverse events than in the placebo groups [12,23].

## 8. Summary and Conclusions

To summarize, there are several observational studies showing an association between vitamin D deficiency and different pain conditions, but a causal relationship is not obvious. Some, but not all, randomized controlled trials have shown positive effect of vitamin D supplementation on pain management. A recent systematic review of published RCTs concluded that vitamin D supplementation leads to a significantly greater mean decrease in pain score compared to placebo in patients with chronic pain. Importantly, patients with sufficient 25-OHD levels from the beginning do not benefit from treatment. The suppression of inflammation in general and of PGE2 especially exhibits credible mechanistic explanations for the effect of vitamin D in pain. Vitamin D supplementation has not shown adverse effects in any studies reviewed here and is also easy to administer. 

Today, the evidence is too weak for making general recommendations for vitamin D in pain management and more randomized and placebo-controlled studies are needed before any firm conclusions can be drawn. However, current knowledge allows us to conclude that patients with deficient levels, defined here as 25-OHD <30 nmol/L, are most likely to benefit from supplementation, while individuals with 25-OHD levels >50 nmol/L probably have little benefit from supplementation. Vitamin D might therefore be offered as independent therapy to vitamin D-deficient patients with chronic pain. Further clinical studies in this field should focus on patients with 25-OHD <30 nmol/L at baseline, and offer intervention that raises the individual’s 25-OHD levels to >50 nmol/L.

## Figures and Tables

**Table 1:** Actions of vitamin D in the immune system that may influence pain management [1,2,6,24,29].

Actions of Vitamin D in the Immune System		
Antimicrobial peptides		**↑**
Inflammation	**↓**	
Th1 cells	**↓**	
Th17 cells	**↓**	
Th2 cells		**↑**
T-regulatory cells (Treg)		**↑**
Prostaglandin E_2_ (PFE_2_)	**↓**	
Tumour Necrosis Factor α (TNF α)	**↓**	
Leukotriene B_4_ (LTB_4_)	**↓**	

T-helper cells (Th); arrow up = increased levels; arrow down = decreased levels.
